# Genomic inbreeding and runs of homozygosity analysis of indigenous cattle populations in southern China

**DOI:** 10.1371/journal.pone.0271718

**Published:** 2022-08-25

**Authors:** Yuqiang Liu, Guoyao Zhao, Xiaojue Lin, Jiahao Zhang, Guanyu Hou, Luepei Zhang, Dewu Liu, Yaokun Li, Junya Li, Lingyang Xu

**Affiliations:** 1 Innovation Team of Cattle Genetic Breeding, Institute of Animal Science, Chinese Academy of Agricultural Sciences, Beijing, China; 2 College of Animal Science, South China Agricultural University, Guangzhou, China; 3 Tropical Crop Germplasm Research Institute, Chinese Academy of Tropical Agricultural Sciences, Hainan, China; Xiamen University, CHINA

## Abstract

Runs of homozygosity (ROH) are continuous homozygous segments from the common ancestor of parents. Evaluating ROH pattern can help to understand inbreeding level and genetic basis of important traits. In this study, three representative cattle populations including Leiqiong cattle (LQC), Lufeng cattle (LFC) and Hainan cattle (HNC) were genotyped using the Illumina BovineHD SNPs array (770K) to assess ROH pattern at genome wide level. Totally, we identified 26,537 ROH with an average of 153 ROH per individual. The sizes of ROH ranged from 0.5 to 53.26Mb, and the average length was 1.03Mb. The average of F_ROH_ ranged from 0.10 (LQC) to 0.15 (HNC). Moreover, we identified 34 ROH islands (with frequency > 0.5) across genome. Based on these regions, we observed several breed-specific candidate genes related to adaptive traits. Several common genes related to immunity *(TMEM173*, *MZB1* and *SIL1*), and heat stress (*DNAJC18*) were identified in all three populations. Three genes related to immunity (*UGP2*), development (*PURA*) and reproduction (*VPS54*) were detected in both HNC and LQC. Notably, we identified several breed-specific genes related to sperm development (*BRDT* and *SPAG6*) and heat stress (*TAF7*) in HNC, and immunity (*CDC23* and *NME5*) and development (*WNT87*) in LFC. Our findings provided valuable insights into understanding the genomic homozygosity pattern and promoting the conservation of genetic resources of Chinese indigenous cattle.

## Introduction

Runs of homozygosity (ROH) are continuous homozygous segments inherited from common ancestors [1]. The size and count of ROH are important factors that reflect potential forces of genomic change. The generation of ROH can be influenced by inbreeding, genetic drift, population bottleneck, as well as natural and artificial selection [2]. Therefore, the detection and characterization of ROH can help to explore population structure and demographic history.

The emergence of high-throughput genotyping technology provided new methods for inbreeding assessment based on single nucleotide polymorphism (SNP). ROH were proposed as a feasible approach to measure the level of inbreeding in livestock [2–5]. The proportion of ROH in autosomal genome can be used to estimate inbreeding coefficient at individual or population levels [6]. The estimation of inbreeding coefficient based on ROH outperformed that of pedigree estimation (because pedigree is often incomplete or inaccurate) [6–8]. Moreover, ROH can be utilized to assess the distribution of homozygous fragments and identify the specific regions with high-frequency ROH on the genome [9].

Domestication and selection can reshape the genomic pattern in many livestock species [10–13]. Strict selection can be achieved by selecting a small number of superior individuals, which can reduce the haploid diversity and increase the frequency of homozygous segments containing favorable genes [14, 15]. ROH can provide valuable insight into the genetic architecture of complex traits [16]. Many studies have been carried out to detect ROH islands and identify a series of genes related to economically important traits in farm animals. For instance, Mastrangelo et al. identified genes related to milk production, immune response and resistance in four Italian cattle breeds [16]. A recent study found that many genes are related to growth, coat color and immunity in different production types of cattle breeds [17]. In local sheep, many genes in ROH islands related to body size and reproduction were found [18–20]. Moreover, several genes related to reproductive traits, meat quality traits and energy conversion were identified within ROH islands in pig [21, 22]. Estimation of Genome-wide mapping inbreeding and the relationship between autozygosity and production traits have widely been explored in dairy and beef cattle [3, 23–26].Moreover, analysis of ROH pattern and their relation to adaptive traits has also been carried out in many Indigenous cattle [27–31].

Indigenous cattle display genetic merits for disease resistance, parasite tolerance, heat tolerance and adapted to local environmental conditions [32]. These cattle can contribute important genetic resources for breeding programs [33]. Understanding the genetic basis underlying adaptive traits can provide valuable resources for global breeding and further help to promote the application of genetic improvement of these cattle [34]. Three indigenous cattle populations are raised in the subtropical regions of southern China (Leiqiong cattle (LQC) and Lufeng cattle (LFC) in Guangdong province, and Hainan cattle (HNC) in Hainan Province). The three breeds are draft, dual-purpure cattle. They show yellow-brown coat color, short straight horns, and small body size. After long-term domestication, these cattle have adapted to the local hot and humid environment, with the merits of strong immunity under rough feeding conditions [35–37].

Investigation of ROH pattern and identification of potentially candidate genes in indigenous cattle populations (LQC, LFC and HNC) living in hot and humid environment conditions are necessary to explain the breed-specific selection in cattle. Despite the ROH pattern of indigenous cattle from China has been explored in our previous analysis [38]. ROH pattern of indigenous cattle population in southern China still remain to be explored. The aims of this work were to (i) evaluate the genome-wide ROH distribution pattern and the inbreeding level of LQC, LFC and HNC using high-density SNP arrays; (ii) identify high-frequency ROH islands across genome and investigate candidate genes in indigenous cattle.

## Materials and methods

### Ethics approval statement

All animals were collected in strict accordance with the Regulations of People’s Republic of China for the Administration of Laboratory Animals (2017 Revision, CLl.2.293192, State Council, China). Animal research protocols were approved by the Institutional Animal Care and Use Committees (IACUCs) of South China Agricultural University (Approval No.2018-P002).

### Genotyped samples

Samples were collected from three cattle populations including Leiqiong (LQC; n = 30), Lufeng (LFC; n = 33) and Hainan (HNC; n = 26), which were genotyped by Illumina BovineHD SNPs array (770K). Genomic DNA was extracted from ear tissue, and DNA with the A260/280 ratio ranging between 1.8 and 2.0 was subject to further analysis. The sample size, associated abbreviation and other information of each population are shown in Table 1. We performed quality control on SNPs array according to the following standards; (i) We removed individuals (PI-HAT value> 0.25) who are closely related as previously reported [38]. (ii) We excluded all SNPs assigned to mitochondrial chromosomes, X and Y, whereas only autosomal SNPs were used in the subsequent analysis. (iii) The individuals with genotype calling rate of more than 99% and SNPs with missing rate less than 5% were kept. (vi) SNPs were also filtered with minor allele frequency (MAF) < 0.05 and genotyping rate (geno < 0.1).

**Table 1:** The descriptive statistics of ROH for three Chinese indigenous cattle populations.

Breed/Population abbreviation	Size	F_HOM_	F_ROH_	F_GRM_	Total ROH number^a^	Average ROH number per individual	Total ROH length (Mb)^b^	Average length of ROH per individual (Mb)
**LQC**	30	-0.06	0.10	0.02	7760	259	7361	245
**LFC**	33	0.08	0.12	0.04	10520	319	10258	311
**HNC**	26	0.10	0.15	0.04	8257	318	9748	375

Note: ^a^. The total number of ROH events for each population.

^b^. The total length of ROH events for each population. LQC: Leiqiong cattle, LFC: Lufeng cattle, HNC: Hainan cattle, F_HOM_: the average inbreeding coefficient based on proportion of homozygous SNP in population, F_ROH_: the average inbreeding coefficient based on proportion of the genome covered by runs of homozygosity in population, F_GRM_: the average inbreeding coefficient based on the genomic relationship matrix.

### ROH estimation

Short ROH can be formed due to linkage disequilibrium across the genome. Therefore, we only detected ROH with a size greater than 0.5Mb [39, 40]. We used PLINK v1.9 to detect ROH across autosomes for each individual [41, 42]. ROH were determined as the following criteria [38]: a sliding window of 50 SNPs across the genome, the proportion of homozygous overlapping windows was 0.05, a minimum number of 100 consecutive SNPs included in a ROH, a maximum gap between consecutive homozygous SNPs of 0.1 Mb, one SNP per 50 k, and maximum two missing SNPs and one heterozygotes genotype in one ROH.

### ROH classification and inbreeding coefficient

ROH were divided into three classes based on size: short (0.5-1Mb), medium (1-5Mb) and large (>5Mb) [38]. We used three methods to evaluate the inbreeding coefficient in the three populations. (i) The proportion of the genome covered by runs of homozygosity (F_ROH_) was estimated based on the total length of ROH divided by the length of autosomes per individual [6]. Moreover, we calculated F_ROH_ per chromosome among the three populations [33]. (ii) compute the F_GRM_ based on genomic relationship matrix(G) as described by previous report [42–44]. We used GCTA v1.19.2 software to calculate the F_GRM_ according to a previous study [45]. (iii) The proportion of homozygous SNP (F_HOM_) based on the observed versus expected number of homozygous genotypes [9, 41, 42].

### Identification ROH island and candidate genes

We conducted a comparison analysis based on the frequency of ROH and identified candidate genes overlapping with ROH segments. In the present study, we defined ROH island based on the consensus overlapping homozygous regions with the frequency higher than 0.50 for each population [46]. In addition, the suggested frequency threshold (0.3) was considered to include more candidate genes. Moreover, candidate genes located within ROH islands were identified based on the reference genome UMD 3.1. [47].

### The distribution of ROH and ROH enriched genes

To investigate the distribution of ROH across population, we estimated the common ROH segments using “—homozyg-group” option implemented in PLINK v1.9. The distributions of ROH were generated using Manhattan plot in R package CMplot (https://github.com/YinLiLin/CMplot). Moreover, the Database for Annotation, Visualization and Integrated Discovery (DAVID) was used to determine Gene Ontology (GO) terms and Kyoto Encyclopedia of Genes and Genomes (KEGG) pathways of candidate genes [48, 49].

## Results

### Genomic ROH distribution

After quality control, 491,515 SNPs and 89 cattle were considered for the downstream analysis. We detected a total of 26,537 ROH in 89 individuals. The size of ROH ranged from 0.5Mb to 53.3Mb in the three populations. Moreover, we observed the highest average number of ROH (319) per individual in LFC, whereas the smallest average number was observed in LQC (259). The largest total lengths of ROH with 375Mb per individual was found in HNC, whereas the smallest with 245Mb was found in LQC. The longest ROH was identified in BTA3 in HNC. Detailed summary statistics of ROH for each population were presented in Table 1.

### ROH pattern and inbreeding coefficients

**Fig 1:**
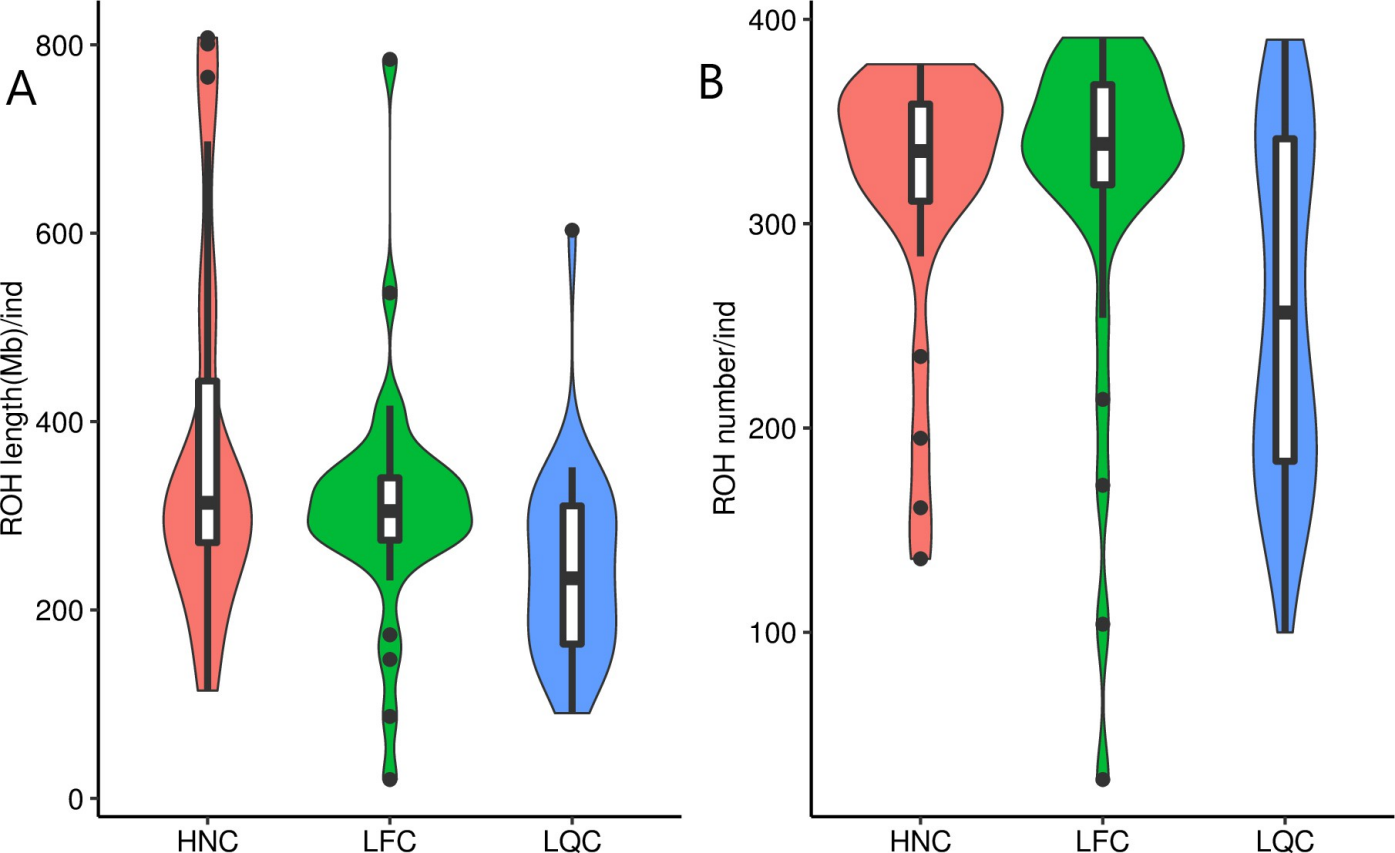
The distributions of ROH statistics per individual for indigenous cattle populations in southern China. (A) Violin plot of the total length of ROH events per individual. (B) Violin plot the total number of ROH events per individual. LQC: Leiqiong cattle; LFC: Lufeng cattle; HNC: Hainan cattle.

**Fig 2:**
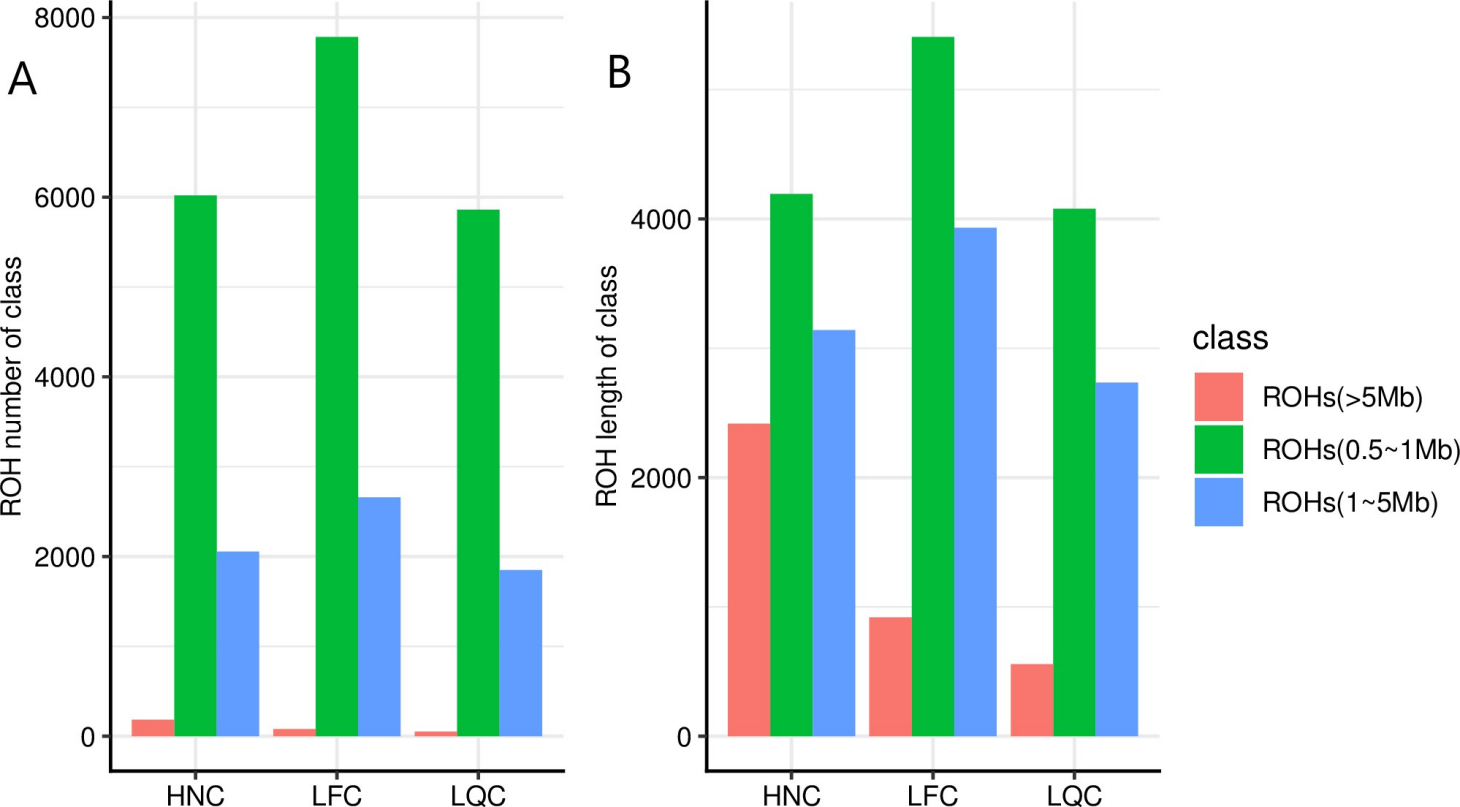
Total length and number of ROH for three size classes including Small (0.5 to 1 Mb), Medium (1 to 5 Mb) and Large (>5 Mb). (A) The total number of ROH for size classes. (B) The total length of ROH for three size classes. LQC: Leiqiong cattle; LFC: Lufeng cattle; HNC: Hainan cattle.

The total ROH length and number for each individual varied among the three populations (Fig 1). Our results showed that HNC contained a large number of long homozygous segments. In contrast, the smallest number and length for ROH was in LQC. To assess the pattern of ROH, we divided ROH into three classes according to their size, as described in previous studies [38]. The distributions of three ROH size classes were presented in Fig 2. We observed proportion of the number of ROH with large length (>5Mb) is 2% in HNC, and 0.7% in LFC and LQC. The total length of the large ROH (>5Mb) was 919Mb, and 556Mb, 2,416Mb in LFC, LQC and HNC, respectively.

**Fig 3:**
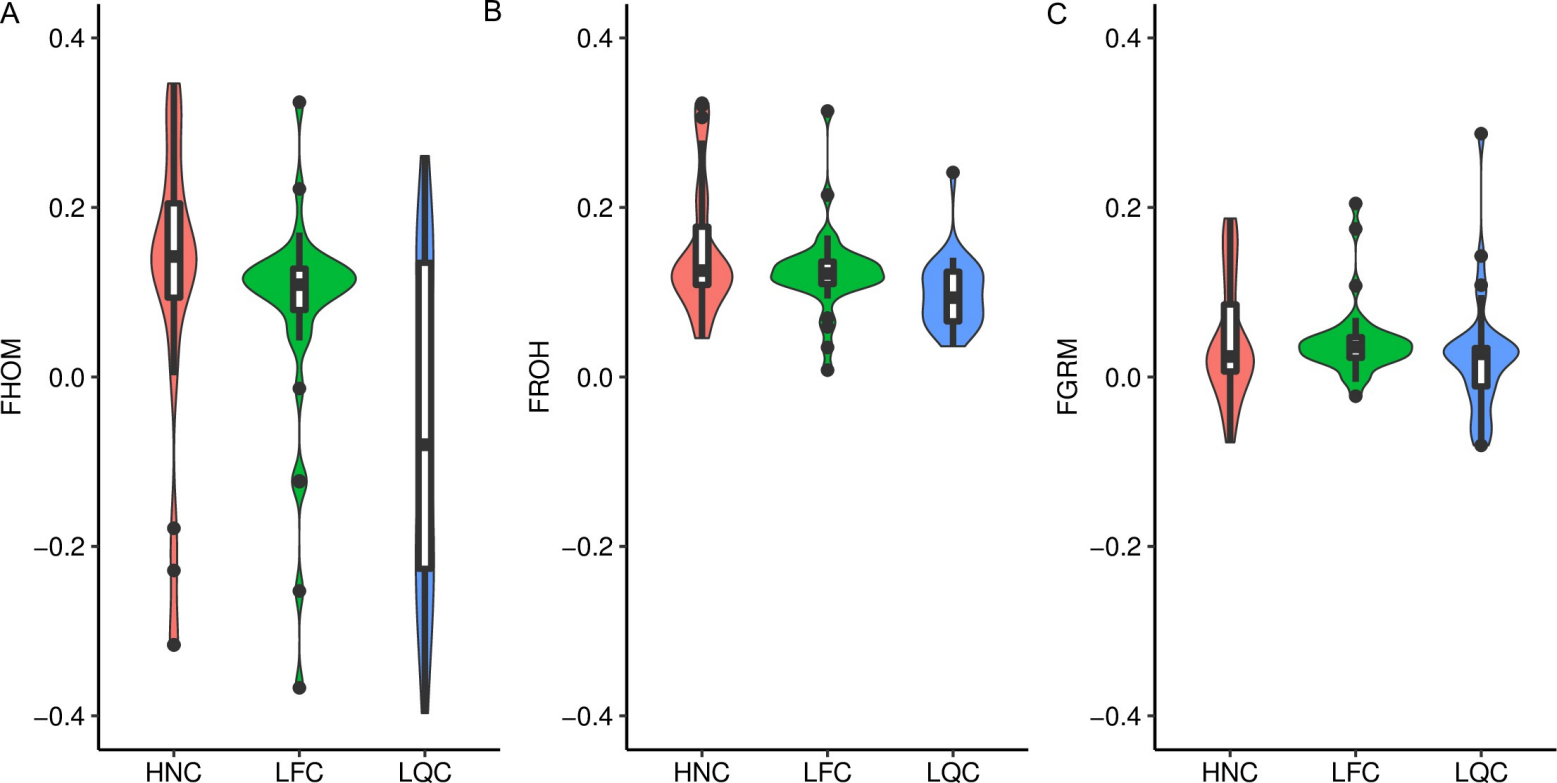
The distributions of inbreeding coefficient. (A) The distributions of F_HOM_ across populations. (B) The distributions of F_ROH_ across populations. (C)The distributions of F_GRM_ across populations. F_HOM_: inbreeding coefficient based on the proportion of homozygous SNP, F_ROH_: inbreeding coefficient based on the proportion of the genome covered by runs of homozygosity, F_GRM_: inbreeding coefficient based on the genomic relationship matrix.

**Fig 4:**
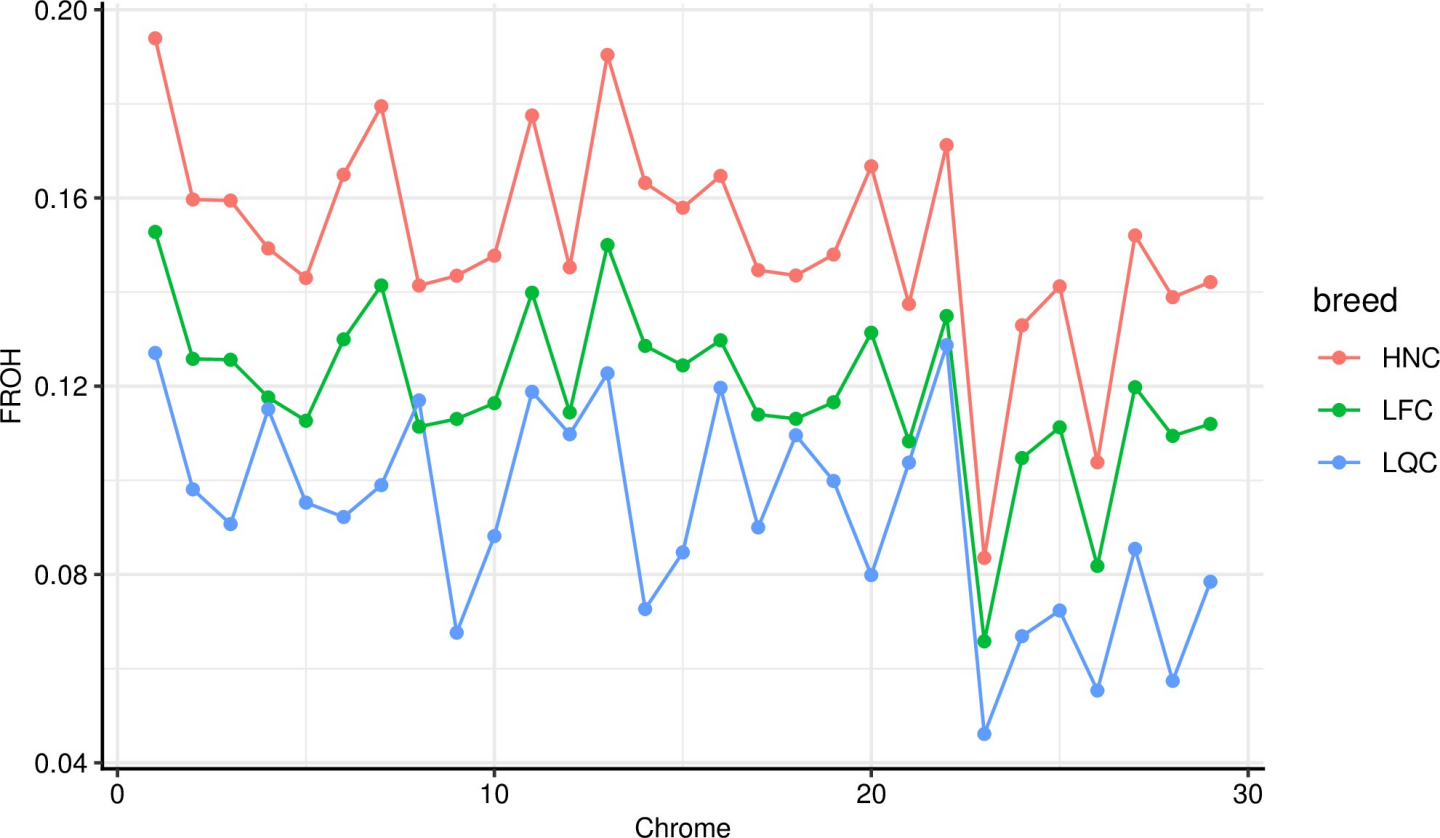
Line plot of inbreeding coefficient for each chromosome among the three populations.

We evaluated inbreeding coefficient using three methods including F_ROH_, F_HOM_ and F_GRM_. We observed the highest F_ROH_ (0.15) in HNC, whereas the minimum (0.10) in LQC. The F_HOM_ ranged from -0.06 to 0.10, whereas the F_GRM_ ranged from 0.02 to 0.04. Using these methods, our results showed similar trend for inbreeding level (Fig 3), and HNC had the highest inbreeding level. Notably, we found F_ROH_ had similar values across 29 chromosomes in HNC and LFC. However, obvious differences were found on BTA4, BTA8, BTA14 and BTA20 in LQC (Fig 4).

### ROH islands distributions across genome

**Fig 5:**
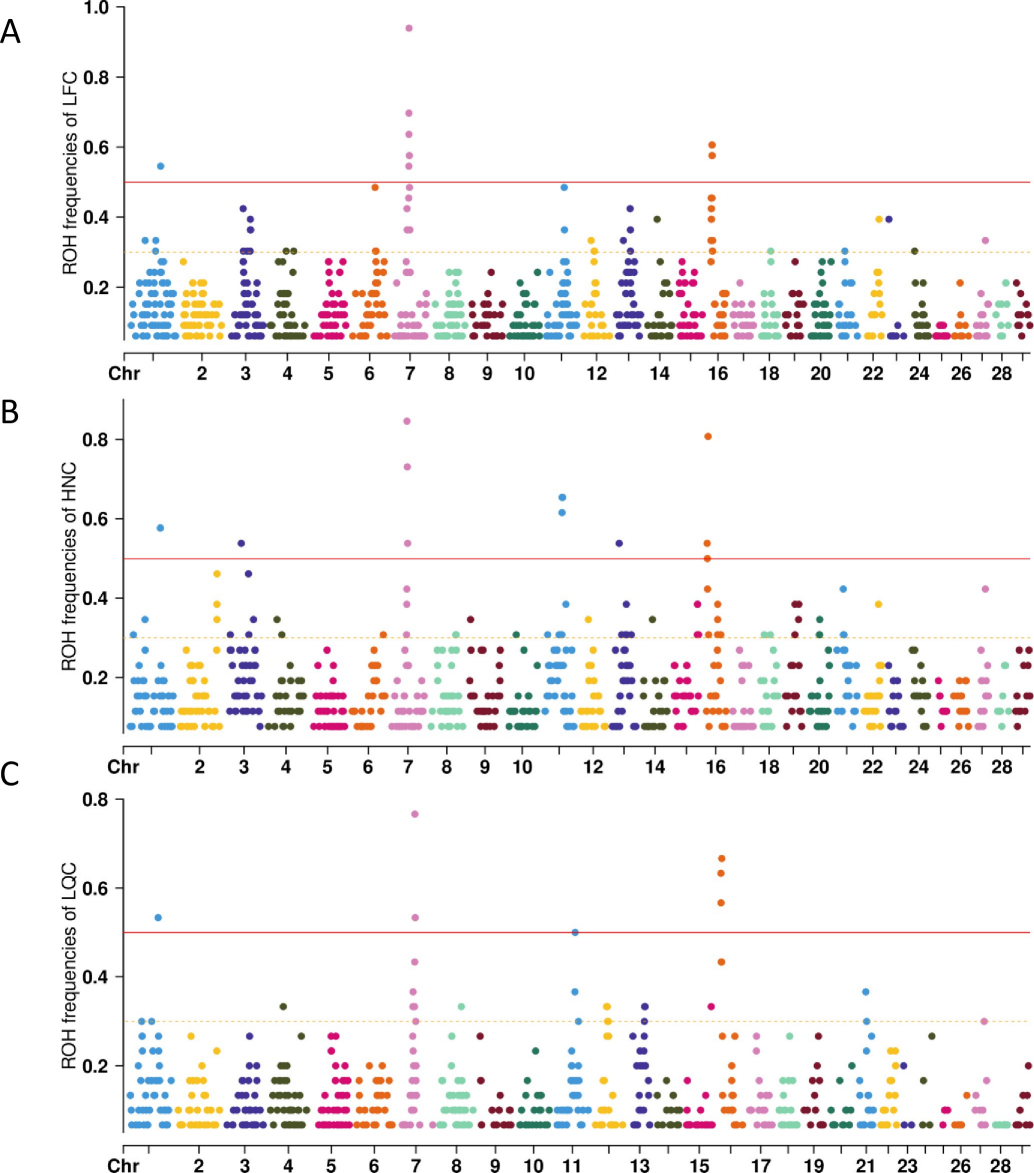
The distribution of ROH across autosomes in the three populations. The x-axis represents the genomic coordinate, and the y-axis displays the frequency of overlapping ROH among individuals. (A) LFC; (B) HNC; (C) LQC.

ROH are suited to detect signatures of selection via ROH islands, we next calculated the frequency of ROH and identified ROH islands for each chromosome. The ROH frequencies of the three populations were presented in Fig 5. We regarded ROH with a frequency larger than 0.5 as ROH islands and searched for candidate genes overlapping with those ROH islands. In total, we identified 7, 11, and 16 ROH islands in LQC, LFC, and HNC, respectively. Notably, we found the shared ROH islands with the highest frequency located at BTA7 in LQC, LFC, and HNC.

### Gene functional annotation

Under the frequency threshold of 0.3, we identified 349 genes based on these ROH islands, and then we performed gene annotation using DAVIDv6.8, we found ten genes (*OMD*, *ITGB8*, *ADAM2*, *PCDHGA8*, *PCDHGC3*, *ITGB3*, *CTNNA1*, *PCDHB11*, *PCDHGA2* and *PCDHGB4*) that were associated to cell adhesion in HNC. Moreover, as for LFC, we found three genes (*ALCAM*, *BSG* and *SEMA3B*) associated to immunoglobulin domain. However, no significant GO term and KEGG pathway were found in LQC. Under the frequency threshold of 0.5, 45 genes were identified based on ROH islands. We observed similar result as to 0.3, and six genes (*PCDHGA8*, *PCDHGC3*, *CTNNA1*, *PCDHB11*, *PCDHGA2* and *PCDHGB4*) associated to cell adhesion in HNC. However, no significant GO term and KEGG pathway were found in LQC and HNC.

### Breed-specific ROH genes

**Fig 6:**
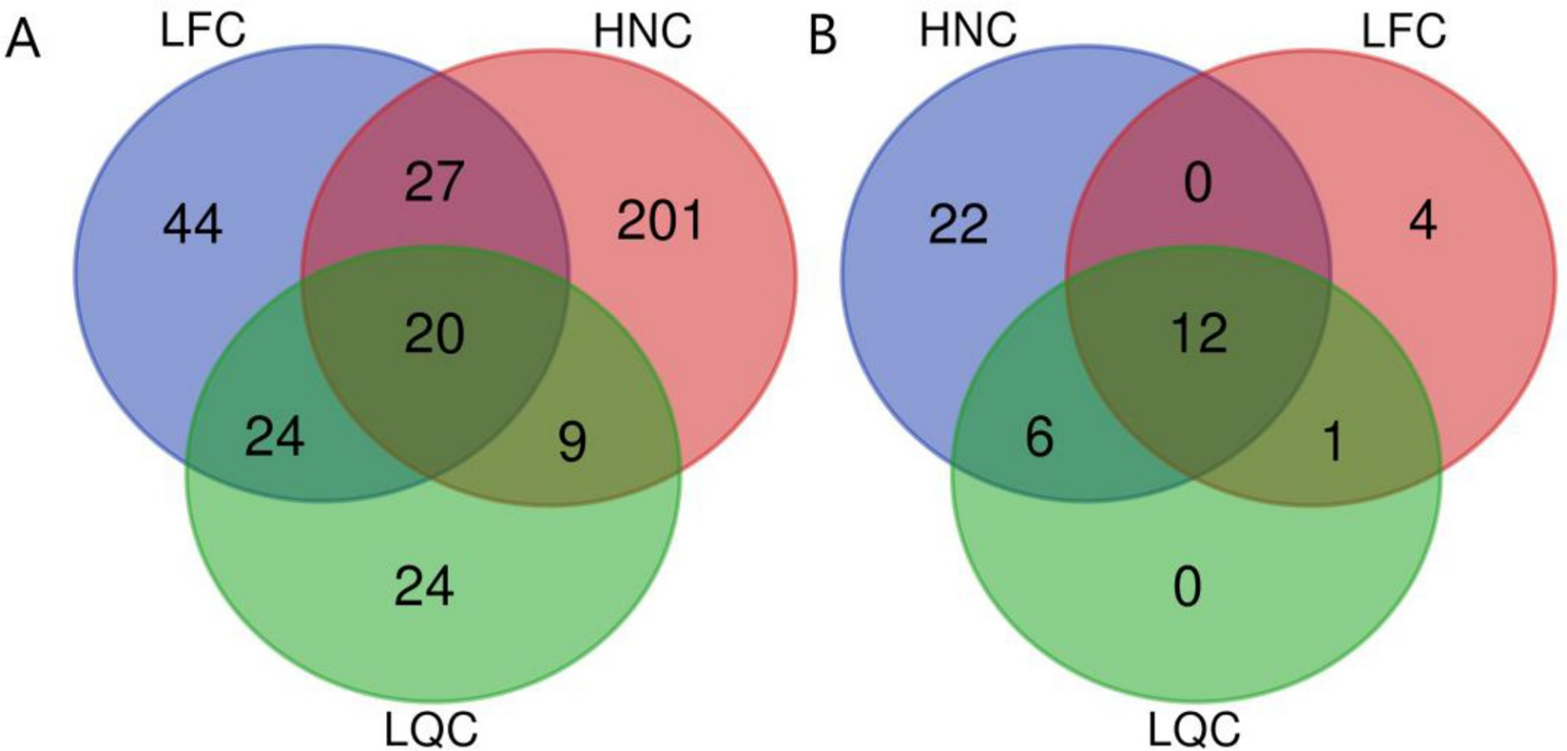
Candidate genes identified for the three populations overlapping with ROH islands. (A) The Venn diagram of the identified genes for three populations when the threshold frequency of ROH islands is set to 0.3. (B) The Venn diagram of the identified genes for three populations when the threshold frequency of ROH islands is set to 0.5.

We identified 19, 17 and 41 genes based on the frequency threshold of 0.5 in LQC, LFC, and HNC, respectively. We found 19 common genes contained at least in two populations, and 26 breed-specific genes (Fig 6). Among them, we identified several common genes related to immunity (*TMEM173*, *MZB1* and *SIL1*), and heat stress (*DNAJC18*) in three populations. Moreover, three common genes were related to immunity (*UGP2*), development (*PURA*) and fecundity (*VPS54*) in HNC and LQC. Notably, we identified several breed-specific genes related to sperm development (*BRDT* and *SPAG6*) and heat stress (*TAF7*) in HNC, and immunity (*CDC23* and *NME5*) and development (*WNT87*) in LFC. Detailed information about high frequency ROH and their related genes were presented in S1 Table.

## Discussion

In this study, we explored the ROH pattern and assessed the inbreeding level in three indigenous Chinese cattle populations using Illumina BovineHD array. Moreover, we identified many breed-specific ROH islands across genome and mapped several candidate genes for important traits.

The distributions of total length and number of ROH implied the genetic differences among populations [50]. Consistent with a previous study [38], we found that the total number and length of ROH were large in indigenous cattle from southern China. Indigenous cattle populations showed a trend that the length and number of ROH increased from north to south [38]. In our study, we found the largest proportion of number and length of large ROH (>5Mb) were identified in HNC (Fig 1). Notably, several individuals with extreme ROH lengths (>800 Mb) were identified among the HNC. As previously reported, the large ROH (>10Mb) was generated during the recent inbreeding (up to five generation ago), whereas short ROH (<1Mb) indicates distant ancestral effect (up to 50 generation ago) [9, 33]. Also, large ROH are likely to be interrupted because of recombination. This finding was consistent with the selection history of HNC [35]. HNC is raised in Hainan province, and the limited genetic introgression occurs from other cattle populations. Thus, the unique environment condition and strict selection prompted the breed formation of HNC. In contrast, LQC had the least number of large ROH (>5Mb), which may be related to genetic introgression from other populations [35]. The mating or cross between outbred individuals or populations may contribute to the disruption of long ROH in the genome [40].

The detection of inbreeding level based on SNP data depends on the actual variation in inbreeding in a population, the effective population size, and the sample size [9]. Mehrnush et al. compared the inbreeding coefficient based on F_PED_, F_GRM_, F_ROH_ and F_TRUE_ (true inbreeding coefficient) in the North American Holstein dairy cattle population and they found that F_ROH_ was closest to the true inbreeding coefficient [51]. We found that HNC had the highest inbreeding level by comparing the inbreeding level of three cattle populations (LFC, LQC and HNC) based on F_PED_, F_GRM_ and F_HOM_. This was agree with previous analysis, and the effective population size of HNC is smaller than that of LQC and LFC [35]. In addition, the inbreeding level of three cattle populations (LFC, LQC and HNC) were higher than that of commercial breeds, whereas similar pattern was observed among them [39, 52–54]. Simultaneously, the high inbreeding coefficient of southern Chinese cattle populations also indicated it is urgent to design feasible mating strategies to control the level of inbreeding and maintain the effective population size for these populations. Moreover, we estimated F_ROH_ for each chromosome in the three populations. HNC and LFC have similar trend on 29 chromosomes, whereas LQC was significantly different in *BTA8*, *BTA14* and *BTA20*. This result can be explained that different selection pressures and recombination occurred may shape breed-specific ROH pattern on different chromosomes [55].

The formation of ROH can be influenced by inbreeding, genetic drift, population bottleneck, recombination events, as well as natural and artificial selection [12, 56]. However, ROH peaks were distributed and shared among individuals, which is likely caused by selection events, demographic history and recombination events [12, 17, 21, 55, 57]. These peaks were called hotspots or ROH islands and can be considered as the signal of selective sweeps across genome. We defined ROH regions with the frequency of more than 0.5 as ROH islands. At last, our analysis identified 34 ROH islands inclusion 45 candidate genes in the three populations. Consistent with the results from previous study [38], we found that many high-frequency ROH islands occur on BTA7 in Chinese local cattle breeds. Moreover, we found that 29 out of 45 overlapped genes.

Among them, we found three genes (*TMEM173*, *MZB1* and *SIL1*) related to immunity and one gene (*DNAJC18*) related to heat stress in all three populations. Transmembrane protein 173 (*TMEM173*) activates the type I interferon-regulated innate immune response, which plays crucial role in modulating inflammation [58]. *MZB1* plays an important role in the process of plasma cell differentiation [59]. Mutations of *SIL1* cause Marinesco-Sjögren syndrome (MSS), which is a neurodegenerative disorder [60]. In a previous study, a member of the heat shock protein family (*DNAJC18*) responding to heat stress has been identified in East African Shorthorn Zebu cattle [61]. These results indicated that the identification of candidate genes for the indigenous cattle populations in southern China may help to explain the genetic basis of adaption for the humid and hot environments. We also found three genes including *UGP2*, *PURA* and *VPS54* related to immunity, reproduction and development in HNC and LQC. *UGP2* plays an essential role in promoting HCC cell migration and tumor metastasis. Mutations in *PURA* may alter normal brain development and impair neuronal function, leading to developmental delay [62]. *VPS54* null mutation may cause embryonic lethality [63].

Notably, we found three breed-specific genes (*WNT8A*, *NME5* and *CDC23*) related to body weight and immunity in LFC. The *WNT8A* contains four single-nucleotides polymorphisms that have an obviously relationship with the height and length of Qinchuan cattle [64]. *WNT8A* was related to the dwarf size in Chinese southern cattle. *NME5* was identified as a candidate gene for primary ciliary dyskinesia and hydrocephalus cases [65]. *CDC23* is a critical regulator of cell cycle and cell growth, and may involve with thyroid cancer initiation and progression [66]. Remarkably, we found three genes (*TAF7*, *SPAG6* and *BRDT*) related to immunity and reproduction in HNC. *TAF7* can regulate the expression of heat shock protein genes and enhance efficient recovery of cells challenged to thermal stress [67]. *SPAG6* acts a crucial role in immuno-regulation, and participate in the occurrence and progression of human cancers. *SPAG6* was also reported that can regulate tumor cell proliferation, apoptosis, invasion, and metastasis [68]. *BRDT* is essential for the normal progression of spermatogenesis, and mutations in *BRDT* can cause male sterility [69]. We suspected that the immune-related genes have been identified among populations, which may reflect the effects of long-term selection for LFC and HNC in the harsh environments.

## Conclusions

In summary, we assessed the ROH pattern, inbreeding level and identified several candidate genes related to important traits in three indigenous cattle populations in southern China. Our findings provided important insights into understanding the genetic basis of adaptive traits and facilitate the protection and breeding management of indigenous cattle population.

## Supporting information

S1 Table The descriptive statistics about the high frequency ROH and candidate genes for indigenous cattle populations in southern China. (XLSX)
